# Neuroimmune Interactions in Inflammation and Acute Kidney Injury

**DOI:** 10.3389/fimmu.2017.00945

**Published:** 2017-08-09

**Authors:** Tsuyoshi Inoue, Shinji Tanaka, Mark D. Okusa

**Affiliations:** ^1^Division of Nephrology, Center for Immunity, Inflammation, and Regenerative Medicine, University of Virginia, Charlottesville, VA, United States

**Keywords:** neuroimmunomodulation, immunity, inflammation, vagus nerve, kidney injury

## Abstract

Inflammation contributes to the pathogenesis of a wide variety of disorders including kidney diseases. Recent advances have shown that neural pathways are able to regulate immunity and inflammation. The cholinergic anti-inflammatory pathway (CAP) is a well-studied neural circuit involving the vagus nerve that is thought to contribute to the response to inflammatory disorders. Expression of receptors for neurotransmitters is found in some immune cells, including β2 adrenergic receptors on CD4 T cells and alpha 7 subunit of the nicotinic acetylcholine (ACh) receptor on macrophages. Once nerves are activated, neurotransmitters such as norepinephrine and ACh are released at nerve terminals, and the neurotransmitters can activate immune cells located in close proximity to the nerve terminals. Thus, vagus nerve stimulation induces activation of immune cells, leading to an anti-inflammatory response. Recent studies demonstrate a non-pharmacological organ protective effect of electrical nerve stimulation, pulsed ultrasound treatment, or optogenetic C1 neuron activation. These modalities are thought to activate the CAP and attenuate inflammation. In this review, we will focus on the current understanding of the mechanisms regarding neuroimmune interactions with a particular focus on inflammation associated with kidney disease.

## Introduction

Recent advances have shown that the communication between the nervous and immune systems can regulate immune function and inflammation (1). The link between the nervous and immune systems depends in part on neural reflexes involving various immune cells. Receptors for neurotransmitters such as acetylcholine (ACh) and norepinephrine (NE) are found on macrophages, dendritic cells, T cells, and B cells (2). These immune cells synthesize and release neurotransmitters and respond to neurotransmitters released from nearby nerve terminals and other immune cells (3, 4). This link between the nervous and immune systems permits rapid homeostatic responses to inflammation and is poised to mediate protection from organ injury. This review will summarize our understanding of the molecular mechanisms that underlie the neural control of inflammation in experimental models of inflammation, mainly focusing on acute kidney injury (AKI) as well as in early studies in human diseases.

## Cholinergic Anti-Inflammatory Pathway (CAP)

A well-studied neuroimmune pathway is the CAP, the efferent limb of the “inflammatory reflex pathway” mediated through the vagus nerve. This concept has been described by Tracey et al. using a lipopolysaccharide (LPS) model of inflammation (1, 5–8). Peripheral vagal afferent neurons express receptors in proximity to immune cells and detect bacterial products (pathogen-associated molecular patterns), proinflammatory cytokines, immunoglobulins and ATP through receptors (9–16). Following detection of these inflammatory molecules, receptors transduce signals from the immune cells and injured tissue and transmit them to the brainstem nucleus tractus solitarius (8, 17). Through uncertain mechanisms vagus efferent nerve is activated and the inflammatory reflex controls peripheral cytokine levels and inflammation.

Mononuclear phagocytes and CD4 T cells are key cellular components that interact to mediate the anti-inflammatory response following activation of CAP. Tracey et al. initially found that direct electrical stimulation of efferent vagus nerve significantly decreased the amount of tumor necrosis factor alpha (TNF-α) in the serum induced by LPS (8). The effect was abolished in mice deficient in the alpha 7 subunit of the nicotinic acetylcholine receptor (α7nAChRKO). α7nAChR is predominantly expressed in neuronal tissues, but Tracey et al. provided histological and functional evidence for α7nAChR on macrophages (11). LPS-induced TNF-α production in peritoneal macrophages was suppressed by ACh or nicotine, an agonist for the nicotinic receptor, and its suppression was abolished in peritoneal macrophages derived from α7nAChRKO mice (18). Downstream of α7nAChR, inhibition of the nuclear translocation of NF-κB (19) and activation of the JAK2–STAT3 pathway (20) ultimately reduces the production of inflammatory mediators.

CD4 T cells importantly contribute to the anti-inflammatory response following activation of CAP. Vida et al. revealed a role for β2 adrenergic receptors on CD4 T cells (21). They showed that the β2 antagonist butoxamine efficiently reversed the TNF-α suppression induced by vagus nerve stimulation (VNS). Furthermore, the anti-inflammatory effect of VNS was lost in β2 adrenergic receptor-deficient mice, but the VNS-induced effect was rescued by the transfer of CD4^+^ CD25^−^ cells (non-T regulatory cells) and not CD4^+^ CD25^+^ cells from WT mice (21). These results provide molecular and pharmacological evidence for the role of CD4^+^ cells in contributing to the anti-inflammatory effect of CAP.

In addition to the importance of macrophages and CD4 T cells, it became evident that the spleen was a crucial organ in the CAP (22). Huston et al. revealed that the spleen is the major source of TNF-α after LPS challenge, and VNS-induced inhibition of systemic TNF-α production was abolished in splenectomized animals (22).

**Figure 1:**
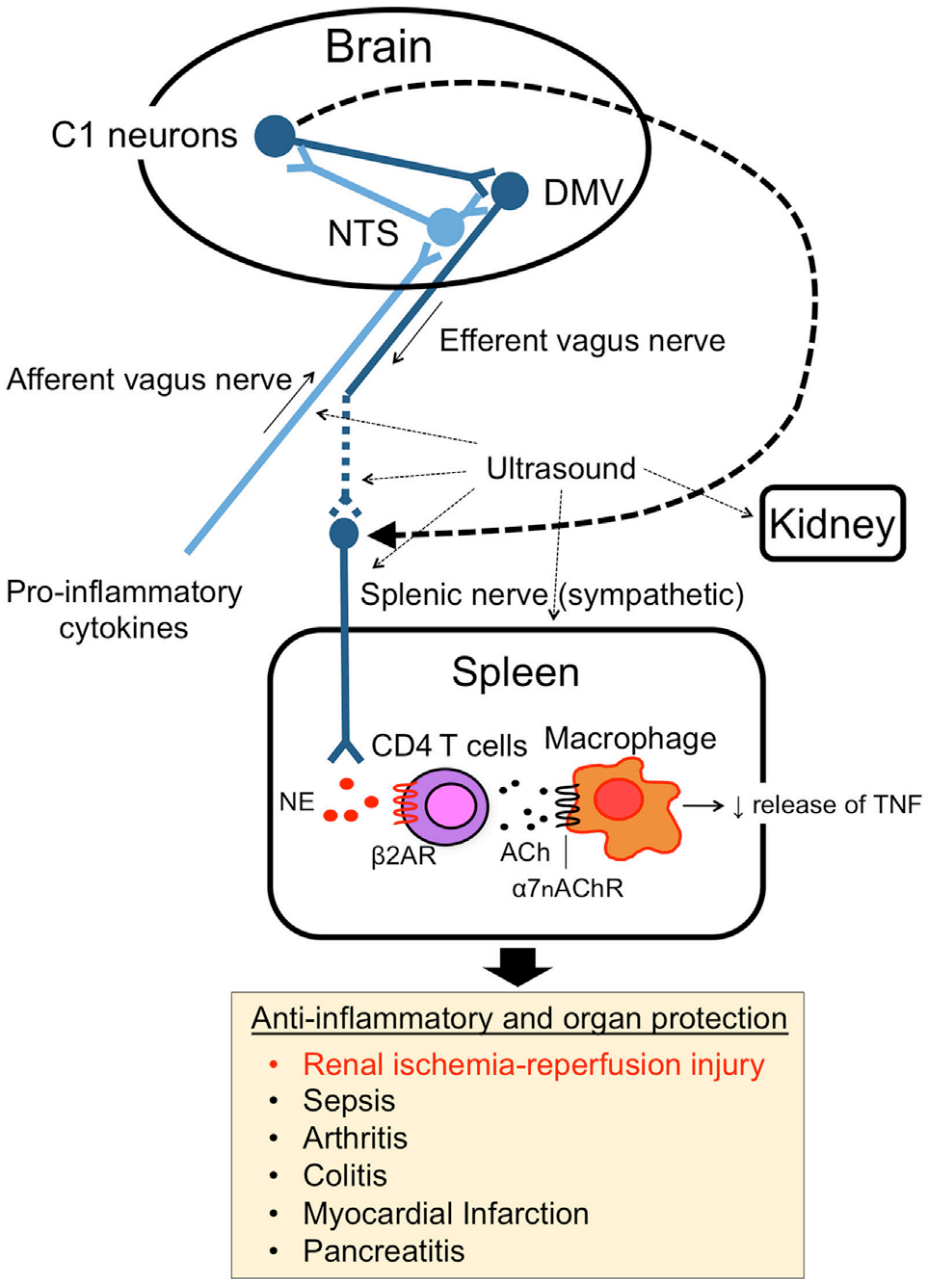
Cholinergic anti-inflammatory pathway (CAP). The CAP links the nervous system and immune system. The activity of afferent vagus nerve fibers is stimulated by cytokines and pathogen-associated molecular pattern molecules. The signal activates efferent vagus nerve fibers through the nucleus tractus solitarius (NTS) and dorsal motor nucleus of the vagus in the brain. The efferent vagus nerve (cholinergic) stimulates CD4 T cells in spleen via the splenic sympathetic (adrenergic) nerve. Release of norepinephrine binds to β2-adrenergic receptors (β2ARs) on CD4 T cells, which then elicits release of acetylcholine (ACh). ACh binding to alpha 7 nicotinic acetylcholine receptors (α7nAChRs) on macrophages produces an anti-inflammatory response, such as TNF-α suppression. Ultrasound and C1 neuron stimulation also activate the pathway and produce organ protective effects.

**Figure 2:**
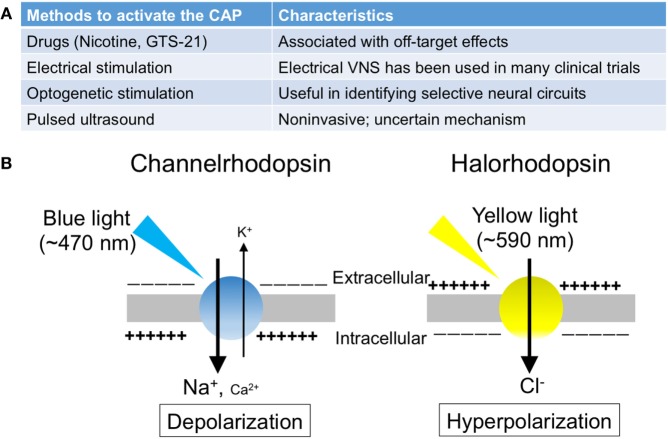
The summary of the methods to activate the cholinergic anti-inflammatory pathway (CAP). **(A)** The list of the methods to activate the CAP. **(B)** Photosensitive ion transporters (used for optogenetic stimulation). When blue (yellow) light is applied to neurons expressing channelrhodopsin (halorhodopsin), the opsin functions as a non-selective cation channel (a chloride pump), resulting in excitation (inhibition) of the neurons.

The splenic nerve, a component of the sympathetic (adrenergic) nervous system, releases NE, whereas the vagus nerve, a component of the parasympathetic (cholinergic) nervous system, releases ACh at nerve terminals upon activation. In response to VNS, ACh in the spleen (3) and NE in plasma (21) are upregulated. Rodent spleen receives noradrenergic fibers from the splenic sympathetic nerves, but little or no direct (cholinergic) innervation from the vagus was found in the spleen (23, 24). Rosas-Ballina et al. elegantly showed that spleen CD4^+^ CD44^high^ CD62L^low^ cells, which express choline acetyltransferase (ChAT) and can release ACh, are the source of ACh in spleen during activation of CAP (3). Catecholaminergic terminals of the splenic nerve in the white pulp of the spleen are in close proximity to lymphocytes, including ChAT-positive T cells, thus permitting functional coupling. The anti-inflammatory effects of VNS are abolished in nude mice, which do not have functional T cells, however reconstituting T cells through adoptive transfer of ACh-synthesizing T lymphocytes into nude mice partially restores VNS-induced anti-inflammatory effect (3). These experiments explain how ACh levels are elevated in the spleen after VNS despite the lack of direct vagus innervation of the spleen. Still unresolved, however, is how NE is released in the spleen (or systemically) after VNS, information that would clarify the interaction between vagus (parasympathetic, cholinergic) and splenic (sympathetic, adrenergic) nerve in CAP. Figure 1 characterizes the CAP based upon current information and Figure 2A summarizes the methods to activate the CAP.

## AKI and Inflammation

Acute kidney injury is a sudden loss of kidney function that occurs within a few hours or up to a few days after an insult and is accompanied by inflammation and tissue damage. AKI causes life-threatening complications such as fluid overload, hyperkalemia, uremia, and metabolic acidosis and can also have systemic effects on other organs including brain, heart, and lung. In hospitalized patients, AKI is a common and major concern because of its high morbidity and mortality (25, 26). In addition, there is a high prevalence of AKI in emergency department patients (27). Various factors such as ischemia, drugs, toxins and infections cause AKI, and inflammation plays a major role in the pathophysiology of AKI including aseptic or sterile AKI (28, 29). The role of immune cells differ depending on the causes of AKI (30). As ischemia–reperfusion injury (IRI) is one of the major causes of AKI and has been studied most extensively, the involvement of both innate and adaptive immunity has been revealed in renal IRI (29–31) as described below.

Following renal ischemia–reperfusion, much of the inflammation occurs in the outer medulla, the part of the kidney with the lowest oxygen tension and with the greatest degree of epithelial cell necrosis. Damage-associated molecular patterns released from dying cells, adhesion molecules, and Toll-like receptors induce the recruitment and activation of various immune cells during the early injury phase (30). Recruitment of neutrophils, natural killer (NK) cells, and NK T cells occurs within several hours of tissue injury, and these cells contribute to initiation of the inflammatory cascade such as IL-1, IL-6, and TNF-α. Leukocyte infiltration into postischemic regions in the kidney is promoted by activation of the complement system in addition to enhanced proinflammatory cytokines and chemokines production (32). Renal dendritic cells increase in number in the injury site and mediate inflammation from the early to late injury phase. Inflammatory monocytes infiltrate the injury site subsequently to neutrophils infiltration and they differentiate into macrophages. In the injury phase, macrophages are polarized into proinflammatory macrophages (M1) and cause tissue damage. By contrast, anti-inflammatory macrophages (M2) predominate during the recovery phase and contribute to resolution of inflammation and tissue repair (31, 33). Tregs play important roles in tubular regeneration, promoting the repair from ischemic injury in addition to their intrinsic renal protective function (34, 35). B cells are activated in the injury phase and limit tubular regeneration in the recovery phase, leading to tubular atrophy (36).

## VNS in Kidney Disease

Inoue et al. evaluated the role of VNS in AKI (37). When VNS was performed 24 h prior to IRI, renal function and tissue morphology were preserved. Plasma TNF-α induction by IRI was also suppressed by prior VNS. Interestingly, this renal protection was observed after either afferent or efferent VNS. Vagus efferents on the opposite side were activated by stimulating vagal afferents on the left side (37). However, the kidney was still protected form IRI by left afferent vagal stimulation with the right vagal nerve blocked with local anesthetic. These findings suggest that afferent VNS and efferent VNS might protect the kidney through different mechanisms. Studies to dissect separate vagal pathways using optogenetic approaches would provide new mechanistic information.

Splenic α7nAChR-positive cells contribute to VNS-induced kidney protection. Splenocytes harvested from WT or α7nAChRKO donor mice 24 h after VNS or sham VNS treatment were transferred into naive WT recipient mice. The recipient mice received renal IRI 24 h later. When the mice received splenocytes from VNS-treated WT mice, the recipient mice were protected from IRI. However, no protection was observed when the recipient mice obtained splenocytes from VNS-treated α7nAChRKO mice (37). The number of macrophages in the kidney increased with time after IRI, but there was no difference in number between WT and α7nAChRKO mice. On the other hand, prior VNS seems to change the polarizing phenotype of macrophages in the kidney. IRI alone induced all M1 markers and most of M2 markers we measured, but suppressed Arg1 expression (an M2 marker) in the infiltrated macrophages in the kidney. Prior VNS rescued Arg1 expression in WT mice, but not in α7nAChRKO mice (37). Thus, VNS-induced protection from kidney injury requires α7nAChR-positive splenocytes and the phenotypic change of macrophages toward M2 might be one mechanism of the protection by VNS.

Inflammation and activation of immune system are also involved in the initiation and development of other kidney diseases such as glomerulonephritis, lupus nephritis, and diabetic nephropathy (38–40). Depending on the phases and types of the kidney diseases, different immune cells, cytokines, and chemokines participate in the pathogenesis of the disease. The involvement of T cells (41) and macrophages (31, 33, 42), key components of the CAP, is reported in various kidney disorders. With rare exception, there are no reports that VNS was applied to the other kidney diseases. VNS was applied to brain dead donors in a model of kidney transplantation (43). VNS stimulation of brain dead donors led to an improvement in transplanted kidney histology and renal function at 16 weeks posttransplantation when compared to unstimulated group. These preclinical data undergird the use of VNS in clinical trials of kidney transplantation. There are limited studies in kidney diseases; however, based on the importance of inflammation and immune responses in these disorders, we believe that many kidney diseases may serve as important therapeutic targets of VNS.

## Ultrasound (US) Protects from AKI Through the CAP

Gigliotti et al. found that prior US application (1 s every 6 s for 2 min on the both sides of kidneys including spleen, mechanical index of 1.2) suppressed systemic and local (renal) inflammation such as IL-6 and TNF-α, and attenuated AKI (44, 45). Experiments in splenectomized mice revealed that the spleen is required for US-induced renal protection (44). US-induced protection was also observed in a cecal ligation and puncture-induced sepsis model (45). Prior US treatment did not protect the kidney in Rag1-deficient mice (Rag1KO) that lack T and B lymphocytes. However, when the Rag1KO were reconstituted with CD4 T cells 10 days prior to US treatment, the protective effect of US was restored (44). In addition, adoptive transfer of splenocytes from US-treated mice 24 h prior to IRI confers protection from IRI (45). These data indicate that CD4 T cells in the spleen are important for US-mediated renal protection.

To investigate whether the protective effects of US were mediated through α7nAChR signaling in CAP, α7nAChRKO mice were used. In α7nAChRKO mice, treatment with US before IRI did not provide protection (44). Bone marrow chimera experiments further revealed the importance of α7nAChRs on hematopoietic cells in US-related organ protection (45). Taken together, these findings suggest that US protects the kidney from AKI through activation of the CAP.

## VNS and Other Neuroimmune Reflexes in Various Disorders

Following initial studies on LPS-mediated inflammation, anti-inflammatory effects of VNS in a wide variety disorders such as arthritis (46), colitis (47), ileus (47), pancreatitis (48), heart disease (49), diabetes (50), and hypertension (51) were confirmed. In contrast to the spleen, the vagus efferent fibers innervate the intestinal wall and directly synapse with postganglionic neurons in the enteric nervous system (52). VNS improves colitis (53) and ileus (54), and vagotomy aggravates these conditions (55). The vagus nerve does not directly innervate the joints; however, VNS attenuated limb inflammation following acute carrageenan-induced arthritis, and vagotomy resulted in increased acute inflammation as evidenced by severe edema (56). Chronic stimulation of vagus nerve, using a method called vagus nerve suspension, improved arthritic scores 3 months after the second immunization in a collagen-induced arthritis model (57). In addition, chronic electrical stimulation of vagus nerve (once daily from day 9 to day 15 for 60 s), using an implanted device with vagus nerve cuff electrodes, reduced the severity of collagen-induced arthritis in rats at day 16 (58).

On the other hand, there are many reports implying the existence of different reflexes and pathways other than the classical CAP. Sciatic nerve activation with electroacupuncture suppresses systemic inflammation following polymicrobial peritonitis through activation of the vagus nerve (59). Stimulation of the sciatic nerve induces vagal-mediated production of aromatic l-amino acid decarboxylase in the adrenal medulla leading to the production of dopamine thereby suppressing systemic inflammation through dopamine type 1 (D1) receptors. Recent studies revealed the importance of sympathetic (adrenergic) nerves (splanchnic and splenic nerves) in a neural reflex pathway that controls inflammation (60). LPS administration strongly increased splanchnic nerve and its splenic branch activity in addition to systemic TNF-α production. LPS-induced increase in plasma TNF-α was enhanced nearly five times when splanchnic nerve was cut, and vagotomy did not affect this. Plasma corticosterone levels were not affected by splanchnic nerve cut, suggesting that in this particular model the neural reflex pathway controlling inflammation was independent of corticosteroids and the vagus nerve. ACh-producing B lymphocytes control local neutrophil recruitment to the peritoneum in response to endotoxin (61). Finally, recent studies suggest a role of β2-adrenergic receptors on the muscularis macrophages, localized deeper in the gut wall (62). Upon luminal bacterial infection, sympathetic (adrenergic) neurons innervating the gut are activated, then muscularis macrophages further enhance tissue-protective programs through β2-adrenergic receptors on the macrophages (62).

## Optogenetic Approaches to Defining the Mechanism of the CAP

Optogenetics is a technique involving the use of light to control the activity of neurons that have been genetically modified to express photosensitive ion transporters (Figure 2B) (63). The discovery of channelrhodopsin-1 (64) and -2 (65) (ChR1 and ChR2) in 2002 and 2003, respectively, marked the genesis of optogenetics. These unique opsins are found in the “eye spot” of *Chlamydomonas reinhardtii*. When blue light (~470 nm) is applied to ChR2, the channel opens and functions as a non-selective cation channel. Only 2 years after the discovery of ChR2, investigators introduced it in mammalian neurons, which were then successfully stimulated using blue light (66).

The utilization of halorhodopsin, an inhibitory channel, gave further impetus to studies using optogenetics (67). When yellow light (~590 nm) is applied to neurons expressing halorhodopsin, the pump allows chloride ions to enter the cells, thereby resulting in hyperpolarization and inhibition of the neurons. In addition, by using the Cre-Lox system, these excitatory or inhibitory proteins can be introduced into specific cell types. Thus, optogenetics enables targeted excitation or inhibition, conferring cellular or projection specificity, which is not attainable through electrical stimulation. Owing to its significant impact on studies in neuroscience, this technique was selected as the “Method of the Year” by the Nature publishing group in 2010 (68).

Because of the many advantages optogenetics provides, this technique is especially useful for revealing the mechanism of the CAP, although it has been underutilized for this purpose thus far. For example, vagal afferent sensory neurons have been divided into several subgroups based on specific markers, and optogenetic stimulation of each subgroup modulated the function of the lung, heart, and gastrointestinal tract in different ways. Selective activation of P2ry1-positive and Npy2r-positive neurons in vagal afferents resulted in apnea and rapid/shallow breathing, respectively (69). It was also reported that Gpr65-positive neurons innervating intestinal villi detect nutrients and regulate gut motility, while Glp1r-positive neurons sense stomach and intestine stretch (70). These findings suggest that nerves in the CAP such as vagus nerve and splenic nerve can be divided into several subgroups and each subgroup might have different roles in the CAP and that optogenetics has the high potential to facilitate elucidation of the selective neural circuits of the CAP.

## C1 Neurons and Restraint Stress in the CAP

By using optogenetics, Abe et al. revealed a new role of C1 neurons in activating the CAP (71). C1 neurons, which reside in the medulla oblongata and innervate the dorsal motor nucleus of the vagus nerve, sympathetic (adrenergic) efferents, the paraventricular nucleus of the hypothalamus, and other brainstem regions, mediate adaptive autonomic responses to several stressors, such as hypotension, hypoxia, and LPS (72). C1 neurons stimulation by laser protects mouse kidneys from IRI. The spleen, β2-adrenergic receptors, and α7nAChRs are necessary for the protective effect, thereby suggesting that the CAP is involved in the protective effect of C1 neuron stimulation. Interestingly, restraint stress for 10 min also protects the kidney from IRI. The protective effect is mediated by C1 neurons and was abolished by ganglionic blockade but not affected by subdiaphragmatic vagotomy or by corticosterone receptor blockade. These findings suggest that C1 neurons activate the CAP not through a vagal, but through a sympathetic (adrenergic) route.

## Vagus Nerve Stimulator

More than 100,000 vagus nerve stimulators have been implanted for medically refractory epilepsy and for treatment-resistant depression and the device has been well tolerated (73). Although transvenous VNS failed to suppress systemic inflammation in healthy subjects (74), pilot studies have showed that implanted vagus nerve stimulators are effective in rheumatoid arthritis (75) and Crohn’s disease (76) in human. In 17 patients with rheumatoid arthritis, including those in early and late stages of the disease, symptoms improved significantly during the period of VNS (75). Biological parameters, including endoscopic index of severity, improved in seven patients with Crohn’s disease and treated with VNS in a 6-month follow-up (76). In addition, many other clinical trials on a wide variety of disorders such as heart failure, hypertension, inflammation, and diabetes are ongoing. Recently two non-invasive external devices have been developed to stimulate the vagus nerve through the skin (73). Transcutaneous cervical VNS reduced the extent of tissue damage after cerebral ischemic injury in rats (77). In humans, VNS with a transcutaneous device on the neck downregulated release of inflammatory cytokines, such as IL-1β and TNF-α, in healthy subjects (78).

## Conclusion

In summary, neuroimmune interactions play very important roles in various disorders, however the mechanisms linking these two different systems are very complex and incompletely understood. Additional studies using advanced tools such as optogenetics will lead to further molecular understanding of neuroimmunomodulatory mechanisms controlling inflammation. These studies could yield potent non-pharmacological approaches to blocking inflammation and tissue injury in systemic disease. In the future, vagus nerve stimulator or US treatment might be applied to the patients who are planned to have cardiac surgery or kidney transplantation, conditions that result from renal ischemia injury.

## Author Contributions

All authors participated in the writing, editing, and literature search. ST was responsible for Figure 2 and TI was responsible for Figure 1.

## Conflict of Interest Statement

The authors declare that the research was conducted in the absence of any commercial or financial relationships that could be construed as a potential conflict of interest.
